# Anti-Skin Aging Potential, Antibacterial Activity, Inhibition of Single-Stranded DNA-Binding Protein, and Cytotoxic Effects of Acetone-Extracted *Passiflora edulis* (Tainung No. 1) Rind Extract on Oral Carcinoma Cells

**DOI:** 10.3390/plants13162194

**Published:** 2024-08-08

**Authors:** Yen-Hua Huang, Cheng-Yang Huang

**Affiliations:** 1 Department of Biomedical Sciences, Chung Shan Medical University, Taichung City 402, Taiwan; 2 Department of Medical Research, Chung Shan Medical University Hospital, Taichung City 402, Taiwan

**Keywords:** *Passiflora edulis*, anti-skin aging, anticancer, Ca9-22 oral carcinoma, tyrosinase, SSB, ssDNA binding, molecular docking, GC–MS analysis, stigmast-5-en-3-ol

## Abstract

The passion fruit, *Passiflora edulis*, recognized for its rich nutritional properties, has long been used for its varied ethnobotanical applications. This study investigates the therapeutic potential of *P. edulis* var. Tainung No. 1 rind extracts by examining their polyphenolic content (TPC), total flavonoid content (TFC), anti-skin aging activities against key enzymes such as elastase, tyrosinase, and hyaluronidase, and their ability to inhibit bacterial growth, single-stranded DNA-binding protein (SSB), and their cytotoxic effects on oral carcinoma cells. The acetone extract from the rind exhibited the highest levels of TPC, TFC, anti-SSB, and antibacterial activities. The antibacterial effectiveness of the acetone-extracted rind was ranked as follows: *Escherichia coli* > *Pseudomonas aeruginosa* > *Staphylococcus aureus*. A titration curve for SSB inhibition showed an IC_50_ value of 313.2 μg/mL, indicating the potency of the acetone extract in inhibiting SSB. It also significantly reduced the activity of enzymes associated with skin aging, particularly tyrosinase, with a 54.5% inhibition at a concentration of 100 μg/mL. Gas chromatography–mass spectrometry (GC–MS) analysis tentatively identified several major bioactive compounds in the acetone extract, including stigmast-5-en-3-ol, vitamin E, palmitic acid, stigmasterol, linoleic acid, campesterol, and octadecanoic acid. Molecular docking studies suggested some of these compounds as potential inhibitors of tyrosinase and SSB. Furthermore, the extract demonstrated anticancer potential against Ca9-22 oral carcinoma cells by inhibiting cell survival, migration, and proliferation and inducing apoptosis. These results underscore the potential of *P. edulis* (Tainung No. 1) rind as a promising candidate for anti-skin aging, antibacterial, and anticancer applications, meriting further therapeutic investigation.

## 1. Introduction

Plants are widely recognized as a rich source of pharmaceutical agents with therapeutic and prophylactic properties, including antioxidant, anti-inflammatory, antimicrobial, and antiproliferative activities [1]. The multifaceted action of natural extracts makes them valuable in therapeutic applications [2]. This underscores the importance of exploring plant extracts for new medical uses [3,4,5,6,7], such as the investigation of *Passiflora edulis* rinds in this study, which looks at their potential to inhibit skin aging and exhibit cytotoxic effects against cancer cells. *P. edulis* [8,9], commonly known as passion fruit, purple granadilla, or egg fruit, belongs to the *Passifloraceae* family, with approximately 60 edible species valued for their economic and medicinal properties. Passion fruit is native to tropical and subtropical regions, consumed fresh or as juice. Traditionally, it has been used in folk medicine for treating insomnia, cough, arthralgia, and constipation [8,9]. The fruit is renowned for its rich nutritional content, including vitamins, dietary fiber, minerals, and a variety of bioactive compounds such as polyphenols, triterpenes, flavonoids, and polysaccharides. Given these attributes, expanding the therapeutic applications of *P. edulis* is compelling.

Skin aging [10] is a complex biological process [11] influenced by intrinsic and extrinsic factors, leading to visible signs such as wrinkles and loss of elasticity [12,13,14,15]. Some natural products could mitigate skin aging by targeting key enzymes involved in the degradation of the skin matrix [16], including tyrosinase, elastase, and hyaluronidase. Prior to this study, very little was known about the anti-skin aging properties of *P. edulis*, a gap this research aims to fill. Tyrosinase, elastase, and hyaluronidase are enzymes critically involved in skin aging, particularly in the degradation of dermal extracellular matrix components. Their overactivity contributes to visible signs of aging, which can potentially be mitigated by incorporating specific inhibitors into cosmetic formulations. This study evaluates the anti-skin aging potential of *P. edulis* (Tainung No. 1) rind extract, focusing particularly on its anti-tyrosinase activity, which plays a crucial role in controlling melanin production.

*P. edulis*, known for its extensive health-promoting properties, has shown potential in various therapeutic contexts, offering antioxidant, anti-inflammatory, antitumor, anti-obesity, hepatoprotective, and neuroprotective benefits [8,9]. This emphasizes the necessity to further explore the functional properties of *P. edulis*, including its various subspecies. Consequently, this study broadens the scope of biological activities examined to encompass anti-skin aging potential, antibacterial activity, inhibition of single-stranded DNA-binding proteins (SSB), and cytotoxic effects against oral carcinoma cells, as investigated using *P. edulis* var. Tainung No. 1. This particular cultivar [17], prevalent in Taiwan, is a hybrid of *P. edulis* (purple passion fruit) and *P. edulis* f. *flavicarpa* (yellow passion fruit), with Nantou County being the central cultivation area, contributing about 75% of Taiwan’s total production. The pulp of this fruit is widely consumed, leaving a significant amount of rind as juice-processing by-product. Typically landfilled or used as compost in local areas, these by-products represent a substantial waste management challenge and potential environmental pollutant. Accordingly, this study also aims to explore extended uses for the rind of *P. edulis* var. Tainung No. 1, potentially transforming a disposal problem into valuable therapeutic applications.

Antimicrobial drug resistance poses an escalating threat to global public health, with cases of antibiotic-resistant bacterial infections rising alarmingly [18,19,20]. Multidrug-resistant pathogenic bacteria are spreading rapidly across the globe, leading to potentially untreatable conditions. For instance, *Staphylococcus aureus* has developed significant antibiotic resistance, contributing to approximately 19,000 deaths annually in the United States alone [21]. Natural plant extracts, known for their antimicrobial properties, are being explored as alternative therapeutic options [22]. Accordingly, this study investigated the antibacterial activity of the acetone-derived rind extract of *P. edulis* var. Tainung No. 1, which showed activity against *S. aureus* and *Pseudomonas aeruginosa*. *P. aeruginosa*, a prevalent opportunistic pathogen, is notorious for causing nosocomial infections and poses significant risks to immunocompromised patients [23]. To date, more than 800 β-lactamases have been identified, with at least 120 detected in *P. aeruginosa* [24], underscoring the urgent need for ongoing development of effective small-molecule antibiotics to combat these antibiotic-resistant pathogens.

SSB is integral to DNA metabolic processes, including replication, repair, recombination, and replication restart in bacteria [25,26,27]. It binds tightly and cooperatively to single-stranded DNA (ssDNA) irrespective of the DNA sequence [28]. SSB also forms interactions with numerous proteins involved in DNA metabolism, collectively known as the SSB interactome [29,30,31,32,33]. Due to its pivotal roles, SSB is considered a potential target for the development of antibacterial drugs [34,35,36]. The Infectious Disease Society of America (IDSA) identifies a group of antibiotic-resistant bacteria known as the ESKAPE pathogens (*Enterococcus faecium*, *Staphylococcus aureus*, *Klebsiella pneumoniae*, *Acinetobacter baumannii*, *Pseudomonas aeruginosa*, and *Enterobacter* spp.), which can effectively “escape” the effects of antibiotics [20,37]. These pathogens are responsible for severe, often lethal diseases and present substantial challenges in treating bacterial infections due to antimicrobial resistance (AMR), contributing to increased morbidity, mortality, and healthcare costs [18,19]. Among these, *K. pneumoniae* is recognized for causing severe hospital- and community-acquired infections, including pneumonia, liver abscesses, and sepsis [38]. This study evaluates the activity of *P. edulis* rind extracts against *K. pneumoniae* SSB (KpSSB), aiming to inhibit DNA replication and reduce the virulence of *K. pneumoniae*, thereby mitigating the threat posed by this dangerous pathogen. The bacterial SSB is structurally and functionally conserved, making KpSSB a valuable model for studying the potential effects on SSBs in other bacterial species.

The widespread occurrence of cancer, particularly oral carcinoma, emphasizes the critical need for innovative therapeutic approaches [39,40,41]. Oral cancer is one of the top ten most common cancers globally, primarily occurring in the oral cavity [42]. Oral squamous cell carcinoma (OSCC), the most common type of head and neck cancer, reports a troubling five-year survival rate of only 50% [43]. Conventional therapies for oral cancers, such as surgery, chemotherapy, and radiation therapy [44], often result in significant side effects and can lead to the development of drug resistance [44]. As a result, there is an increasing interest in natural compounds as supplementary anticancer agents alongside traditional treatments [1]. This study focuses on the cytotoxic effects of *P. edulis* (Tainung No. 1) rind extract, extracted with acetone, on Ca9-22 gingival carcinoma cells. Demonstrating high total phenolic and flavonoid contents, the rind extract from *P. edulis* var. Tainung No. 1 has shown potential in preclinical settings for its ability to induce apoptosis and inhibit cell proliferation and migration, crucial factors in effective cancer management.

In this study, we explored the biological properties of *P. edulis* extract, focusing on its potential for anti-skin aging, antibacterial activity, SSB inhibition, and cytotoxic effects against oral carcinoma cells. Additionally, gas chromatography–mass spectrometry (GC–MS) was employed to tentatively identify the chemical constituents of the extract. The seven most prevalent compounds were further analyzed through molecular docking with both tyrosinase and KpSSB to computationally investigate potential inhibition mechanisms. Overall, our findings underscore the potential novel therapeutic applications of *P. edulis* rinds, warranting further scientific exploration into their medical uses.

## 2. Results

### 2.1. Anti-Skin Aging Potential

Skin aging is a universal process that ultimately affects all individuals [10,11]. Prior to this study, very little was known about the anti-skin aging properties of *P. edulis*. In this study, we examined rind extracts from *P. edulis* (Tainung No. 1) for their potential to mitigate skin aging. The evaluation was based on the extracts’ inhibitory effects on key enzymes associated with aging, specifically tyrosinase, elastase, and hyaluronidase. Using known inhibitors as positive controls—epigallocatechin gallate for anti-elastase, kojic acid for anti-tyrosinase, and myricetin for anti-hyaluronidase—the inhibitory activity of the rind was assessed using solvents 100% methanol, ethanol, and acetone (Table 1). The extracts demonstrated significant anti-aging enzyme inhibition at 100 μg/mL, with the highest observed effects being 54.5% inhibition for tyrosinase and 18.4% inhibition for hyaluronidase by acetone-extracted rind. While anti-tyrosinase and anti-hyaluronidase activities were found, these three extracts did not exhibit an anti-elastase effect at the used concentration of 100 μg/mL. For the first time, we found the rind extract from *P. edulis* (Tainung No. 1) capable of inhibiting the activity of tyrosinase significantly.

### 2.2. Antibacterial Activity

The antibacterial properties of various *P. edulis* (Tainung No. 1) rind extracts, obtained using water, methanol, ethanol, and acetone, were evaluated using the agar-well diffusion method [45]. Antibacterial efficacy was measured by the diameter of the inhibition zones (Table 2), targeting human pathogens such as *Escherichia coli*, *Staphylococcus aureus*, and *Pseudomonas aeruginosa*. The extracts (10 mg) exhibited varying levels of antibacterial activity, with inhibition zones ranging from 5 to 17 mm. Notably, the acetone extract exhibited the most potent antibacterial activity against all three tested organisms. Among the extracts, the antibacterial effectiveness of the acetone-extracted rind was ranked in the following order: *E. coli* > *P. aeruginosa* > *S. aureus*.

### 2.3. Total Phenolic Content (TPC)

Polyphenols [46] are increasingly acknowledged for their potential as cosmeceuticals and pharmaceutical agents, underscored by rigorous validation screens [47,48,49,50]. Accordingly, we evaluated the total phenolic content (TPC) of *P. edulis* (Tainung No. 1) rind extracts, utilizing the modified Folin–Ciocalteu method to quantify TPC (Table 3). The analysis revealed that the acetone-extracted rind exhibited the highest TPC, registering 4.1 mg GAE/g.

### 2.4. Total Flavonoid Content (TFC)

Flavonoids are natural compounds known for their diverse structure-dependent biological and pharmacological activities [51]. Therefore, we also determined the total flavonoid content (TFC) of *P. edulis* (Tainung No. 1) rind extracts (Table 4). TFC was quantified using the aluminum chloride colorimetric method. The analysis revealed that the acetone-extracted rind exhibited the highest TFC, registering 5.4 mg RUE/g.

### 2.5. Gas Chromatography–Mass Spectrometry (GC–MS) Analysis

Given that the acetone-extracted rind extract of *P. edulis* (Tainung No. 1) exhibited the highest TPC, TFC, and the highest anti-tyrosinase and anti-hyaluronidase activities among these rind extracts, we further conducted gas chromatography–mass spectrometry (GC–MS) analysis to tentatively identify the predominant compounds within the extract (Figure S1). The spectral data generated were compared with the NIST 2011 and Wiley 10th edition mass spectral libraries, allowing for the tentative identification of the compounds. Compounds with a similarity index (SI) greater than 800 were considered for identification. Accordingly, the top 13 compounds, each constituting more than 0.5% of the extract, were identified as follows: stigmast-5-en-3-ol (10.3%), vitamin E (7.5%), palmitic acid (5.6%), stigmasterol (3.0%), linoleic acid (2.9%), campesterol (2.3%), octadecanoic acid (2.0%), heptacosane (1.0%), sitostenone (1.0%), eicosane (0.7%), squalene (0.7%), pentacosane (0.6%), and docosane (0.5%). Although HPLC–MS analysis previously identified many constituents in *P. edulis* [52], the compounds identified through this GC–MS study could enrich the existing knowledge of *P. edulis’*s chemical profile.

### 2.6. Molecular Docking Analysis of Tyrosinase

**Figure 1:**
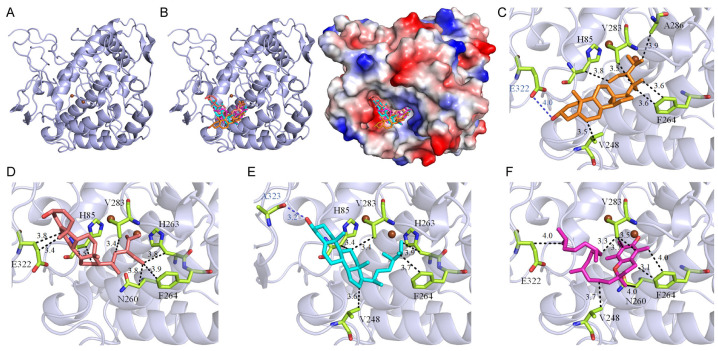
Molecular docking analysis of tyrosinase. (**A**) The crystal structure of tyrosinase (PDB ID 2Y9X) is shown, with the complexed molecule 2-hydroxycyclohepta-2,4,6-trien-1-one removed for clarity. Active site copper ions are highlighted in brown. (**B**) Docking analysis demonstrating that four compounds from the extract exhibit higher binding affinities than kojic acid: campesterol (cyan), stigmasterol (orange), stigmast-5-en-3-ol (deepsalmon), and vitamin E (light magenta). These compounds have the capacity to dock into the tyrosinase active site, potentially obstructing substrate access and inhibiting enzyme activity through various binding poses. (**C**–**F**) Binding modes of stigmasterol, stigmast-5-en-3-ol, campesterol, and vitamin E with tyrosinase, illustrating their interactions within the enzyme’s active site. The blue dashed lines indicate hydrogen bonds, and the black dashed lines indicate hydrophobic interactions. The numbers labeled on the dashed lines represent the distances in angstroms.

Tyrosinase, a crucial copper-containing enzyme, regulates melanogenesis, impacting skin conditions like age spots, photodamage, and pigmentation [53,54]. In this study, the acetone-extracted rind of *P. edulis* (Tainung No. 1) exhibited potent inhibitory activity against tyrosinase (Table 1). Following this, compounds from the extract were analyzed through GC–MS and subjected to molecular docking simulations using AutoDock Vina to evaluate their binding affinities to tyrosinase (PDB ID 2Y9X) [55], predicting potential inhibition mechanisms (Table 5). The tropolone originally bound within the complex was removed prior to docking (Figure 1A). Seven prominent compounds from the extract were docked into tyrosinase’s active site (Figure 1B), showing binding energies ranging from −5.0 kcal/mol to −7.8 kcal/mol, with stigmasterol displaying the highest affinity. Kojic acid, a recognized tyrosinase inhibitor, was also docked for comparison, exhibiting a binding energy of −6.1 kcal/mol. A lower binding energy suggests a more stable interaction with the enzyme, indicating that stigmasterol, stigmast-5-en-3-ol, campesterol, and vitamin E may have the highest affinity for tyrosinase among the tested compounds, potentially surpassing even kojic acid (Table 5). The hierarchy of binding efficiency for these compounds, relative to kojic acid, was established as follows: stigmasterol > stigmast-5-en-3-ol, campesterol > vitamin E > kojic acid > linoleic acid > palmitic acid, octadecanoic acid. Each compound’s docking into the active site potentially blocks substrate access, thus inhibiting tyrosinase activity through diverse binding poses (Figure 1B and Table 6). Stigmasterol demonstrated substantial interactions within the active site of tyrosinase (Figure 1C), forming a hydrogen bond with Glu322 (4.0 Å) and engaging in hydrophobic interactions with His85 (3.8 Å), Val248 (3.5 Å), Phe264 (3.6 Å), Val283 (3.5 Å), and Ala286 (3.9 Å). Stigmast-5-en-3-ol exhibited hydrophobic interactions with His85 (3.5 Å), Asn260 (3.8 Å), His263 (3.8 Å), Phe264 (3.9 Å), Val283 (3.4 Å), and Glu322 at distances of 3.4 Å and 3.8 Å (Figure 1D). Campesterol (Figure 1E) formed a hydrogen bond with Ala323 (3.2 Å) and engaged in hydrophobic contacts with His85 (3.4 Å), Val248 (3.6 Å), His263 (3.9 Å), Phe264 (3.7 Å), and Val283 (3.4 Å). Vitamin E (Figure 1F) interacted hydrophobically with Val248 (3.7 Å), Asn260 (4.0 Å), Phe264 at both 3.1 Å and 4.0 Å, and Val283 at 3.3 Å, 3.5 Å, and 3.7 Å, alongside Glu322 (4.0 Å). These varied molecular interactions suggest a combined inhibitory mechanism exerted by these compounds on tyrosinase. However, further biochemical and structural studies are required to substantiate these preliminary findings.

### 2.7. Inhibition of SSB by the Extract of P. edulis

**Figure 2:**
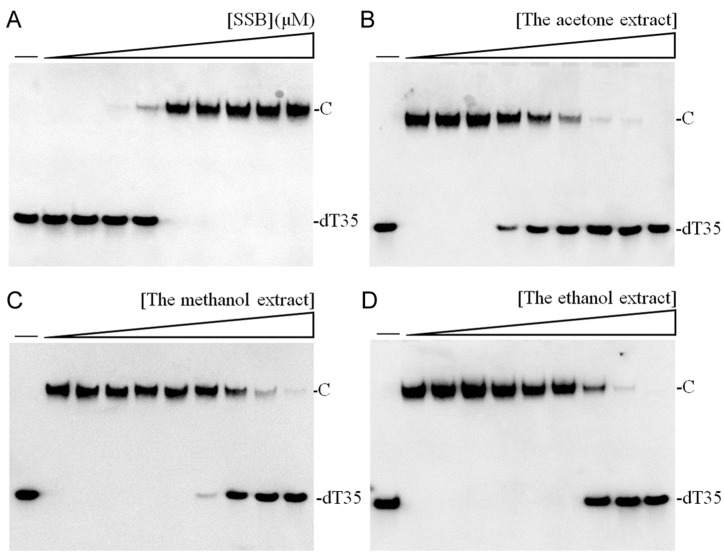
Inhibition of SSB by *P. edulis* rind extracts. (**A**) SSB binding to ssDNA. SSB from *K. pneumoniae* across a range of concentrations (0, 18, 37, 77, 155, 310, 625, 1250, 2500, and 5000 nM) was incubated with a biotinylated dT35 oligonucleotide. A streptavidin–horseradish peroxidase conjugate was used to detect the ssDNA and the resulting complexes. C indicates the formed complex. (**B**–**D**) Inhibition of ssDNA-binding activity of SSB by *P. edulis* rind extracts obtained using acetone (**B**), methanol (**C**), and ethanol (**D**). SSB (310 nM) was treated with varying concentrations of each extract (0, 31, 63, 125, 250, 500, 1000, 2000, and 3000 μg/mL) to assess inhibition of binding activity.

The acetone-extracted rind of *P. edulis* demonstrated notable antibacterial activity (Table 2). Consequently, SSB, essential for DNA replication, repair, and recombination [26,32,36,56], was targeted to evaluate the extract’s potential as an antimicrobial agent. *Klebsiella pneumoniae* SSB [57,58] served as the model protein for this analysis. The recombinant SSB was purified via Ni^2+^-affinity chromatography. An electrophoretic mobility shift assay (EMSA) was conducted to determine the binding affinity of SSB to ssDNA at varying protein concentrations [59,60]. A biotinylated deoxythymidine (dT) homopolymer, dT35, was utilized as the substrate to assess SSB binding, characterized by a decrease in electrophoretic mobility upon protein binding. The assay results, visualized through a streptavidin–horseradish peroxidase conjugate, indicated a band shift signifying the formation of SSB–DNA complexes. Complete binding was observed at a concentration of 310 nM SSB (Figure 2A). From the titration data, the binding constant ([Protein]_50_) was determined to be 205.9 ± 14.3 nM (Figure 2A). Further, the inhibition of SSB activity by the acetone-extracted rind was investigated by introducing the extract at concentrations ranging from 31 to 3000 μg/mL. The EMSA results demonstrated that SSB binding to ssDNA was inhibited by the extract at concentrations between 125 and 3000 μg/mL (Figure 2B). Through the titration curve, an IC_50_ value of 313.2 ± 21.0 μg/mL was determined, indicating the potency of the acetone extract in inhibiting SSB (Table 7). Water-extracted rinds at concentrations of 3000 μg/mL did not influence the activity of SSB. Comparative assays with methanol-extracted (Figure 2C) and ethanol-extracted rinds (Figure 2D) also showed inhibitory effects on SSB, though less potent than the acetone extract. This suggests that specific compounds within the acetone extract of *P. edulis* rind, either individually or synergistically, could serve as effective anti-SSB agents.

### 2.8. Molecular Docking Analysis of SSB

**Figure 3:**
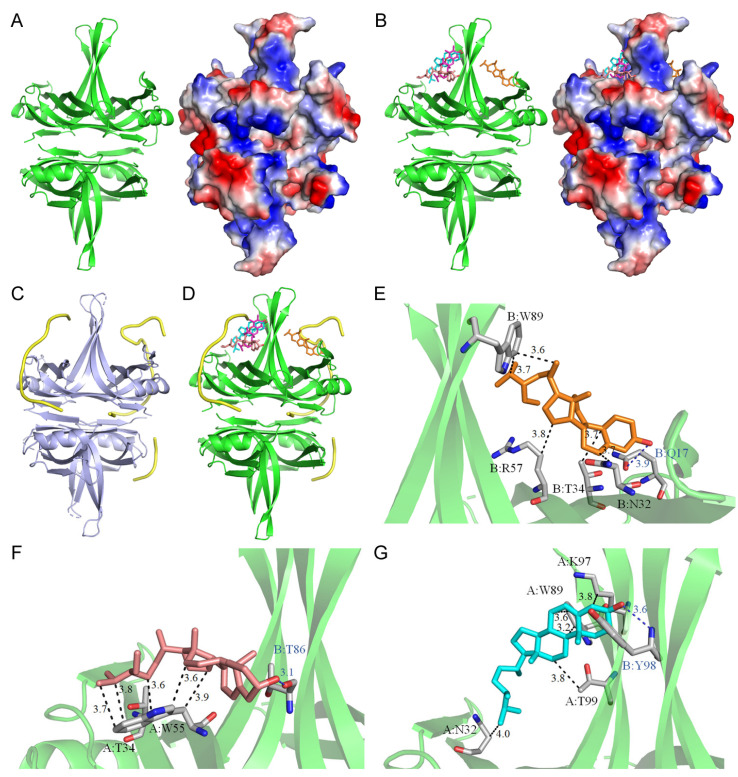
Molecular docking analysis of SSB. (**A**) Crystal structure of *K. pneumoniae* SSB. *K. pneumoniae* SSB is a homotetramer. The charge distribution pattern is shown for clarity to indicate possible binding sites. (**B**) Docking analysis depicting the four most prevalent compounds from the extract, each individually docked into SSB: stigmasterol (orange), stigmast-5-en-3-ol (deepsalmon), campesterol (cyan), and vitamin E (lightmagenta). (**C**) The structure of *P. aeruginosa* SSB bound by ssDNA dT20. The ssDNA within the complex crystal structure of the *P. aeruginosa* SSB tetramer is highlighted in yellow. Given the unavailability of an ssDNA-complexed structure for *K. pneumoniae* SSB, the complexed structure of the *P. aeruginosa* SSB is utilized for comparative analysis of the ssDNA-binding mode in *K. pneumoniae* SSB. (**D**) Superimposed structures of ssDNA bound by *P. aeruginosa* SSB alongside the docked compounds bound by *K. pneumoniae* SSB, suggesting potential ssDNA-binding sites in *K. pneumoniae* SSB. (**E**–**G**) Binding modes of stigmasterol (**E**), stigmast-5-en-3-ol (**F**), and campesterol (**G**) to *K. pneumoniae* SSB are illustrated, highlighting their interactions within the binding sites.

Due to their high inhibitory potential (Table 7), the predominant compounds in the acetone-extracted rind of *P. edulis* were evaluated to elucidate their potential mechanisms of inhibition via molecular docking studies. Following the identification through GC–MS analysis, the seven primary compounds were docked into the *K. pneumoniae* SSB using AutoDock Vina, which showed binding energies ranging from −4.2 kcal/mol to −6.8 kcal/mol (Table 5). Four compounds—stigmasterol, stigmast-5-en-3-ol, campesterol, and vitamin E—demonstrated significant binding affinities (<−6 kcal/mol) and were further analyzed for their interaction modes (Table 8). Previously, we solved the crystal structure of *K. pneumoniae* SSB (Figure 3A), which is a homotetramer [57]. This structure (PDB ID 7F2N) was used for our docking analysis (Figure 3B), displaying varied binding poses. Due to the absence of an ssDNA-complexed structure for this SSB, the structure of *Pseudomonas aeruginosa* SSB complexed with ssDNA dT20 (PDB ID 6JDG) [61,62] was used as a comparative model (Figure 3C). Given their structural similarities, the ssDNA-binding modes between these two SSBs are presumed to be comparable (Figure 3D). Docking results indicate that these four compounds may competitively occupy ssDNA-binding sites, potentially disrupting the ssDNA-binding activity of SSB (Figure 3D). Stigmasterol forms a hydrogen bond with Gln17 and extensive hydrophobic interactions with Asn32, Thr34, Arg57, and Trp89 of SSB (Figure 3E). Stigmast-5-en-3-ol creates a hydrogen bond with Thr86 and engages in significant hydrophobic interactions with Thr34 and Trp55 (Figure 3F). Campesterol establishes a hydrogen bond with Tyr98 while forming extensive hydrophobic contacts with Asn32, Trp89, Lys97, and Thr99 (Figure 3G). Considering the diverse interactions, including those involving vitamin E at various ssDNA-binding sites, the inhibitory efficacy of the *P. edulis* extract against SSB may result from the synergistic effects of these compounds. However, these preliminary findings necessitate further biochemical and structural validation.

### 2.9. Cytotoxic Effects of Acetone-Extracted P. edulis Rind

**Figure 4:**
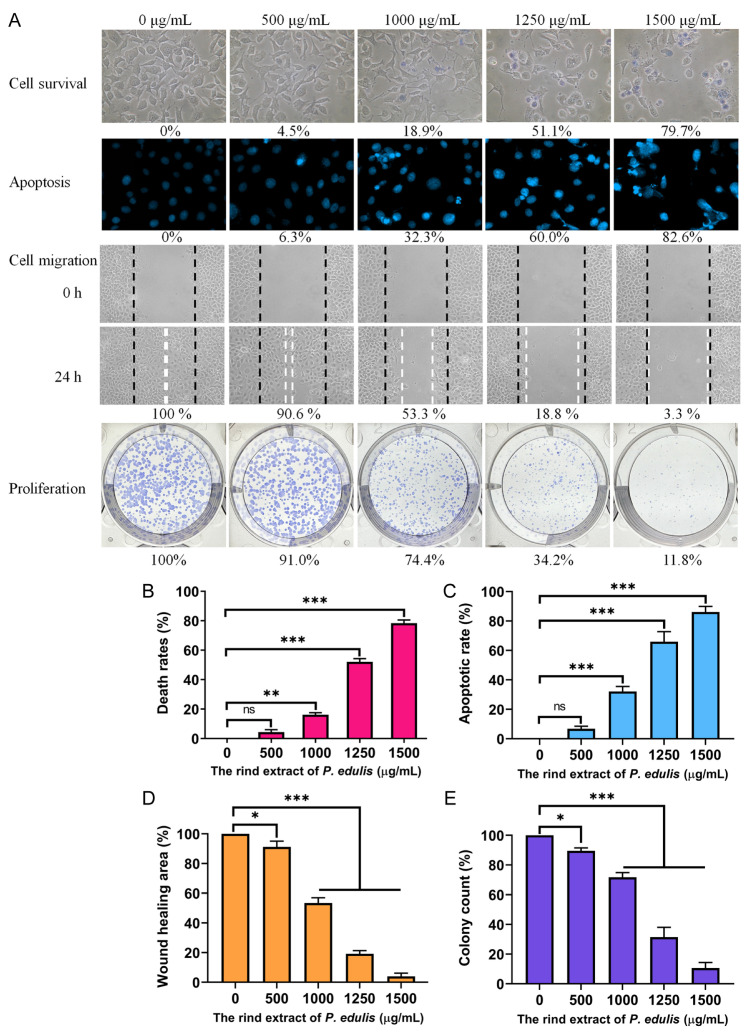
Anticancer potential of acetone-extracted *P. edulis* rind on Ca9-22 gingival carcinoma cells. (**A**) Overview of the rind extract’s influence on Ca9-22 cell viability, migration, proliferation, and nuclear condensation. (**B**) Results from the Trypan blue exclusion assay, illustrating cell viability post-treatment with varying concentrations of the rind extract. (**C**) Hoechst staining analysis depicting the extent of apoptosis and DNA fragmentation across different concentrations of the rind extract. (**D**) Wound healing assay images capturing Ca9-22 cell migration before and 24 h post-extraction treatment at various concentrations. (**E**) Clonogenic assay results evaluating the ability of Ca9-22 cells to form colonies under different concentrations of the rind extract, reflecting their survival and proliferative capabilities. Statistical significance relative to the control is indicated by * for *p* < 0.05, ** for *p* < 0.01, and *** for *p* < 0.001. A control medium containing 1% DMSO served as the negative control and caused deaths at the rate of 0%, reduced migration by 0%, suppressed proliferation and colony formation by 0%, and did not induce apoptosis in Ca9-22 cells.

Globally, cancer remains a significant cause of mortality, with incidence rates continuing to rise. Notably, polyphenols, which are prevalent in various natural products, have demonstrated notable anticancer properties. Accordingly, this study also assessed the cytotoxic potential of acetone-extracted rind from *P. edulis* (Tainung No. 1), which exhibited high TPC and TFC, against cancer cells (Figure 4A). The evaluation focused on various parameters, including cell viability, migration, proliferation, and DNA fragmentation—a hallmark of apoptosis. Oral cancer, consistently listed among the ten most common cancers worldwide, primarily develops in the oral cavity. In this investigation, Ca9-22 gingival carcinoma cells were used as a model. Cell viability was determined using the trypan blue exclusion assay (Figure 4B), which relies on the principle that viable cells do not absorb the dye due to their intact membranes, whereas non-viable cells do. This assay facilitates the distinction and enumeration of live versus dead cells. Treatments with the rind extract were prepared from a stock solution of 20 mg/mL and adjusted with the culture medium. Controls included cells cultured in medium containing 1% DMSO. Dose–response effects were observed, with extract concentrations of 500, 1000, 1250, and 1500 μg/mL inducing cell death rates of 4.5%, 18.9%, 51.1%, and 79.7%, respectively. These findings suggest that the acetone-extracted rind of *P. edulis* (Tainung No. 1) possesses promising anti-oral cancer properties.

The human normal cell line HEK293T was also used for the cytotoxicity assay to evaluate the anticancer potential of the extract. We found that treatment with extract concentrations of 0, 1000, and 1250 μg/mL did not induce death in HEK293T cells (Figure S2). Accordingly, the cytotoxic effects of the acetone-extracted *P. edulis* rind may exhibit specificity; however, this should be further confirmed through additional cellular, biochemical, and clinical studies.

### 2.10. Induction of Apoptosis in Ca9-22 Cells by Acetone-Extracted P. edulis Rind

The acetone extract from the rind of *P. edulis* (Tainung No. 1) was found to effectively induce apoptosis in Ca9-22 cells. Apoptosis was confirmed using Hoechst 33342 staining, a technique where the dye penetrates all cells but exhibits increased fluorescence upon binding to the condensed chromatin typical of apoptotic cells. Quantitative assessment of DNA fragmentation, a definitive marker of apoptosis, indicated significant increases in response to the extract at various concentrations: 500, 1000, 1250, and 1500 μg/mL resulted in 6.3%, 32.3%, 60.0%, and 82.6% increases in DNA fragmentation, respectively (Figure 4C). Morphological changes characteristic of apoptosis, such as chromatin condensation and nuclear fragmentation, were observed to intensify with increasing concentrations of the extract, demonstrating a dose–response relationship in the induction of apoptotic cell death. The prominent blue fluorescence of the Hoechst dye, upon binding to apoptotic nuclei, further verified these apoptotic characteristics. Consequently, these results suggest that bioactive compounds within the acetone-extracted rind of *P. edulis* activate apoptotic pathways, elucidating their mechanism of action and underscoring the extract’s potential as a therapeutic agent against cancer.

### 2.11. Inhibition of Ca9-22 Cell Migration by Acetone-Extracted P. edulis Rind

The acetone extract derived from the rind of *P. edulis* (Tainung No. 1) exhibited significant anti-migratory effects on Ca9-22 cells. These effects were quantified using a wound healing assay, a well-established in vitro technique that evaluates collective cell migration on a two-dimensional plane (Figure 4D). The assay involves the creation of a cell-free zone within a confluent monolayer to mimic a wound, prompting the adjacent cells to migrate and close the gap as part of a simulated tissue repair process. Quantitative analysis showed that the application of the extract at concentrations of 500, 1000, 1250, and 1500 μg/mL resulted in reductions in cell migration of 9.4%, 46.7%, 81.2%, and 96.7%, respectively, over a 24-h period. These findings validate the extract’s substantial efficacy in inhibiting the migratory capabilities of oral carcinoma cells, suggesting its potential role in the prevention of cancer metastasis.

### 2.12. Inhibition of Ca9-22 Cell Proliferation by Acetone-Extracted P. edulis Rind

The effects of the acetone extract from the rind of *P. edulis* (Tainung No. 1) on the proliferation of Ca9-22 cells were investigated using a clonogenic assay, which measures the ability of individual cells to grow into colonies, thus reflecting their survival and proliferative capabilities (Figure 4E). The assay demonstrated a dose-dependent inhibition in colony formation, indicating significant antiproliferative activity. Specifically, the treatment with the extract at concentrations of 500, 1000, 1250, and 1500 μg/mL led to reductions in colony formation by 9.0%, 25.6%, 65.8%, and 88.2%, respectively. These findings highlight the potential of the extract to decrease the viability and proliferation of oral carcinoma cells, effectively limiting their capacity for progression and dissemination.

## 3. Discussion

*Passiflora*, commonly referred to as “passion fruit”, thrives in tropical and subtropical climates [9]. This fruit is consumed both fresh and as an ingredient in processed foods. Extracts, juices, and individual compounds derived from passion fruit exhibit diverse health benefits and biological activities, including antioxidant, anti-inflammatory, sedative, and neuroprotective properties [8,9]. Accordingly, exploring new therapeutic uses for this fruit is highly warranted. Furthermore, waste from fruits and vegetables, such as the rinds utilized in this study, not only represents a loss of valuable resources but also poses environmental challenges. Efficiently reducing and repurposing food waste to create value-added products can enhance production processes and decrease associated costs. In this study, we discovered that the rind of *P. edulis* (Tainung No. 1), extracted with acetone, exhibited high TPC (Table 3) and TFC (Table 4), along with notable anti-skin aging (Table 1), antibacterial (Table 2), and anti-oral cancer potentials (Figure 4). This study is the first to evaluate the anti-SSB (Table 7 and Figure 2) and anti-skin aging properties (Table 1) of *P. edulis*. GC–MS analysis identified the top 13 compounds in this extract, shedding light on the active components potentially responsible for these biological activities and their possible synergistic interactions. Although HPLC–MS analysis previously identified many constituents in *P. edulis* [52], the compounds identified through this GC–MS study could enrich the existing knowledge of *P. edulis’*s chemical profile. Thus, valorizing waste products such as passion fruit rinds is of considerable interest for their added value.

In vitro studies consistently underscore the health-promoting effects of phytochemicals [63,64]. The skin, as the body’s largest and most intricate organ, plays a vital barrier role against external influences [11]. Its condition greatly affects social perceptions, with youthful and healthy skin typically regarded more favorably [15]. Aging alters skin structure and function, leading to thinness, dryness, decreased elasticity, rough texture, wrinkles, and dark spots. These changes can significantly affect overall health and quality of life. Hence, the continuous discovery of anti-aging agents is crucial [65]. Plant-derived compounds [14], especially secondary metabolites and whole plant extracts, have been thoroughly investigated for their anti-aging benefits [10]. Polyphenols, known for their potent antioxidant properties, help mitigate aging and photodamage [66]. Specific compounds such as quercetin and myricetin are recognized for their ability to inhibit enzymes like tyrosinase and hyaluronidase, which are vital in the skin aging process [67]. This study demonstrates the anti-skin aging potential of *P. edulis* (Tainung No. 1), particularly the acetone-extracted rind extract, which exhibits significant inhibition of tyrosinase. These insights highlight the necessity of further research into specific bioactive compounds within the extract that target aging-associated enzymes, particularly tyrosinase, to develop effective anti-aging therapies.

Our findings indicate that the acetone-extracted rind extract of *P. edulis* (Tainung No. 1) exhibits strong inhibitory effects on tyrosinase, suggesting potential expanded therapeutic uses. Tyrosinase, a crucial copper-containing enzyme in melanogenesis, facilitates the conversion of L-tyrosine to L-DOPA and its subsequent oxidation to dopaquinone, thereby regulating melanin production [68]. Recent studies have linked tyrosinase activity not only to age-related skin changes such as spots, photodamage, and pigmentation but also to the development of Alzheimer’s disease [69,70]. This neurodegenerative condition is marked by cognitive deterioration, memory loss, and behavioral changes, with its pathology associated with amyloid-beta deposition, neurofibrillary tangles, oxidative stress, a deficit in cholinergic function, and neuroinflammation [71]. Tyrosinase’s byproduct, L-DOPA, is implicated in neurotoxic effects, inflammatory responses, and increased tau protein phosphorylation [72]. Additionally, heightened tyrosinase activity, particularly in the substantia nigra, contributes to neuromelanin production [73]. Given the considerable side effects associated with current Alzheimer’s treatments, targeting tyrosinase inhibition could represent a dual-purpose therapeutic approach, addressing both skin aging and the progression of Alzheimer’s disease. All seven major compounds from the extract successfully docked into the active site of tyrosinase (Figure 1). These compounds demonstrated binding energies ranging from −5.0 kcal/mol to −7.8 kcal/mol, with stigmasterol exhibiting the highest affinity at −7.8 kcal/mol, surpassing even kojic acid, a well-known tyrosinase inhibitor, which displayed a binding energy of −6.1 kcal/mol (Table 5). Additionally, stigmast-5-en-3-ol, campesterol, and vitamin E from the extract also exhibited higher affinities for tyrosinase compared to kojic acid, highlighting the potential of the *P. edulis* (Tainung No. 1) rind extract for drug development. The cooperative action of these compounds (Table 6), by individually docking into the enzyme’s active site and obstructing substrate access, suggests a collective mechanism that inhibits tyrosinase activity through varied binding poses. Therefore, the potent inhibitory effects of *P. edulis* (Tainung No. 1) rind extract on tyrosinase warrant further investigation into its potential as a source of tyrosinase inhibitors for applications in both dermatological and neurotherapeutic domains.

Although acetone is less safe compared to ethanol as an extraction solvent, it remains effective for extracting natural products. For example, using acetone as the solvent has proven beneficial for enhancing TPC (Table 3) and TFC (Table 4). For future applications of the acetone-extracted *P. edulis* (Tainung No. 1) rind extract, it is crucial to ensure the complete volatilization of acetone from the extract.

In this study, we explored the acetone-extracted rind of *P. edulis* (Tainung No. 1) and discovered its potential to inhibit the activity of SSB in *K. pneumoniae* (Figure 2). SSB is crucial for DNA replication and cellular survival, highlighting its significance as a target for anti-pathogenic therapies [34,57,74,75]. *K. pneumoniae* is noted for its resistance to antibiotics and is one of the ESKAPE pathogens [20,23,76], which can effectively “escape” the effects of conventional antibiotics. The inhibition of essential proteins like SSB offers a promising avenue for developing new antimicrobial strategies [77,78]. Targeting DNA replication and repair mechanisms has been a foundational approach in antibiotic development, exemplified by the success of quinolones and aminocoumarins, which inhibit bacterial DNA gyrase and topoisomerase IV [79,80]. Given the profound antibacterial properties of various plant extracts, continuing the search for effective SSB inhibitors is of significant interest. Our findings underscore the potential of *P. edulis* (Tainung No. 1) extract as a therapeutic agent against bacterial infections by targeting this critical DNA replication protein (Table 8 and Figure 3). Currently, our laboratory is investigating specific bioactive compounds within the extract that could serve as potent antibacterial agents. Additionally, the correlation between the extract’s toxicity against oral carcinoma cells and its inhibition of ssDNA-binding activity merits further investigation.

The acetone-extracted rind of *P. edulis* (Tainung No. 1) exhibited cytotoxic properties against Ca9-22 oral carcinoma cells, manifesting through inhibition of cell migration and proliferation and induction of apoptosis (Figure 4). The GC–MS analysis tentatively identified key compounds within the extract, including stigmast-5-en-3-ol, vitamin E, palmitic acid, stigmasterol, linoleic acid, campesterol, octadecanoic acid, heptacosane, sitostenone, eicosane, squalene, pentacosane, and docosane. The primary constituents, notably stigmast-5-en-3-ol [81], vitamin E [82], palmitic acid [83], stigmasterol [84,85], linoleic acid [86], campesterol [84,87], are recognized for their anticancer activities. Future investigations should focus on the synergistic and polypharmacological effects of these bioactive molecules, aiming to determine the most effective combinations and concentrations for cancer therapy.

## 4. Materials and Methods

### 4.1. Materials

The chemicals used in this study were sourced from Sigma-Aldrich (St. Louis, MO, USA). Ca9-22 gingival carcinoma cell lines were obtained from the Food Industry Research and Development Institute in Hsinchu, Taiwan [77]. These cells were cultured as monolayers in Dulbecco’s Modified Eagle Medium (Gibco™; Thermo Fisher Scientific, Waltham, MA, USA), supplemented with 10% fetal bovine serum (FBS), 100 units/mL penicillin, and 100 μg/mL streptomycin. The cells were incubated at a constant 37 °C in an atmosphere of 95% air and 5% CO_2_.

### 4.2. Plant Materials and Extract Preparations

Fresh fruits of *P. edulis* (Tainung No. 1) were sourced from a private farm in Nantou County, Taiwan [17]. After cleaning to remove impurities and checking for any damages, the fruit rinds were prepared for extraction. The rinds were dried, finely chopped, and ground into powder. For the extraction process, 1 g of this powder was placed in a 250 mL conical flask along with 100 mL of an extracting solvent (water, methanol, ethanol, or acetone). This mixture was agitated on an orbital shaker for 5 h. The resulting extract was filtered through a 0.45 μm filter, and the solvent was subsequently removed in a hot air circulation oven set at 40 °C. The dry extracts were stored at −80 °C until use. For experimental applications, the extract was dissolved in 20% DMSO to prepare a stock solution of 20 mg/mL. For anticancer assays, this stock solution was further diluted with supplemented culture medium to achieve the desired experimental concentrations. Cancer cells were then treated with these dilutions or with a control medium containing 1% DMSO.

### 4.3. GC–MS Analysis

The rind extract of *P. edulis* (Tainung No. 1), obtained through acetone extraction, was characterized using gas chromatography–mass spectrometry (GC–MS). The analysis employed a Thermo Scientific TRACE 1300 Gas Chromatograph coupled with a Thermo Scientific ISQ Single Quadrupole Mass Spectrometer System. Chromatographic separation was facilitated using a Rxi-5ms column (30 m × 0.25 mm i.d. × 0.25 μm film), with helium as the carrier gas at a flow rate of 1 mL/min. The oven temperature program began at 40 °C, held for 3 min, then ramped at 10 °C/min to a maximum of 300 °C, where it was maintained for 1 min. The injection port was consistently set at 300 °C. Detection was conducted using a quadrupole mass detector with electron ionization, featuring operational settings of a quadrupole temperature of 150 °C, a source temperature of 300 °C, electron energy of 70 eV, a detector temperature of 300 °C, an emission current multiplier voltage of 1624 V, and an interface temperature of 300 °C. The mass range scanned was 29 to 650 amu. Relative mass fractions of the detected chemical components were calculated using peak area normalization. Compounds were tentatively identified by comparing the generated spectra against the NIST 2011 and Wiley 10th edition mass spectral libraries, with those showing a similarity index (SI) above 800 considered positively identified and reported in this study.

### 4.4. Determination of TPC

The quantification of TPC was carried out using the modified Folin–Ciocalteu method [88]. The absorbances of the blue color developed were measured at 750 nm by using a UV/VIS spectrophotometer (Hitachi U 3300, Hitachi High-Technologies, Tokyo, Japan) [89]. Gallic acid (GAE) at varying concentrations served as the positive control. The results for the extracts were benchmarked against the standard curves of GAE and are expressed in mg equivalent per g of dry weight of the plant material. Values represent the mean standard deviation from three independent experiments.

### 4.5. Determination of TFC

The quantification of TFC was carried out using the aluminum chloride colorimetric method [90]. The absorbance of extracts and standard solutions was measured at 510 nm by using a UV/VIS spectrophotometer (Hitachi U 3300, Hitachi High-Technologies, Tokyo, Japan). Rutin at various concentrations was employed as the positive control. The outcomes from the extracts were evaluated against the standard curves of rutin and are reported as mg equivalent per g of dry weight of the plant material. The values presented are the mean standard deviation derived from three independent experiments.

### 4.6. Tyrosinase Inhibition

The tyrosinase inhibitory activity was evaluated using a spectrophotometric method based on the oxidation of 3,4-dihydroxy-L-phenylalanine (L-DOPA), employing a modified dopachrome method [91,92]. Extracts were initially solubilized in 20% DMSO and subsequently diluted to the desired concentrations using 0.1 M phosphate buffer (pH 6.8). Each reaction mixture, prepared in a 96-well microtitre plate, consisted of 5 μL of the extract, 115 μL of 0.1 M phosphate buffer, 40 μL of mushroom tyrosinase (200 units/mL), and 40 μL of L-DOPA (2.5 mM). The mixtures were incubated at 37 °C for 30 min. For background absorbance correction, a blank was included for each sample, containing all the reaction components except L-DOPA. The absorbance was measured at 475 nm. Kojic acid served as a positive control, demonstrating expected inhibition, while the negative control contained 10% DMSO instead of the extract. The percentage of tyrosinase inhibition was calculated with the formula: Inhibition % = [(*A*_control_ − *A*_sample_)/*A*_control_] × 100.

### 4.7. Hyaluronidase Inhibition

The inhibition of hyaluronidase activity was quantified using a protocol adapted from existing literature [93]. Each assay was initiated by pre-incubating 25 μL of the test extract with 3 μL of hyaluronidase from bovine testes type I-S (H3506, Sigma-Aldrich, USA) diluted to 0.4 U/mL in a 20 mM phosphate buffer (pH 7.0), which also contained 77 mM sodium chloride and 0.01% bovine serum albumin (BSA). This mixture was incubated at 37 °C for 10 min. Subsequently, 12 μL of 300 mM phosphate buffer (pH 5.35) were added, followed by an additional 10-minute incubation at 37 °C. Then, 10 μL of a hyaluronic acid substrate solution (0.03% *w*/*v* in 300 mM phosphate buffer, pH 5.35) was introduced, and the reaction mixture was incubated for 45 min at 37 °C to facilitate the breakdown of hyaluronic acid. The reaction was terminated by the addition of 100 μL of acidic albumin solution (24 mM sodium acetate, 79 mM acetic acid, and 0.1% BSA, pH 3.75), and allowed to stabilize at room temperature for 10 min. Absorbance was then measured at 600 nm. Myricetin was employed as a positive control, and 10% DMSO served as the negative control. The degree of hyaluronidase inhibition was calculated using the following formula: Inhibition % = [(*A*_control_ − *A*_sample_)/*A*_control_] × 100.

### 4.8. Elastase Inhibition

The elastase inhibition assay was performed using a modified protocol from prior research [94]. The reaction was set up in 200 mM Tris–HCl buffer (pH 8.0). Porcine pancreatic elastase was prepared at a concentration of 3.33 mg/mL in the same buffer. The substrate N-Succinyl-Ala-Ala-Ala-p-nitroanilide (AAAPVN) was solubilized at 1.6 mM in 200 mM Tris–HCl. The test extracts were pre-incubated with elastase for 15 min to allow for interaction. The complete reaction mixture, totaling 250 μL per well, included the buffer, 0.8 mM AAAPVN, 2 μg/mL elastase, and the test extract. Epigallocatechin gallate was used as a positive control, and 10% DMSO served as the negative control. The absorbance was measured at 405 nm immediately following the addition of the substrate and monitored over a 20-minute period in a 96-well plate format. The percentage of elastase inhibition was determined using the following formula: Inhibition % = [(*A*_control_ − *A*_sample_)/*A*_control_] × 100.

### 4.9. Trypan Blue Cytotoxicity Assay

Cell viability was evaluated using the trypan blue exclusion assay to determine the cytotoxic effects of the extract on Ca9-22 cells [95,96]. Cells were seeded at a density of 10,000 cells per well and exposed to the seed extract in a total volume of 100 μL for 24 h. This assay facilitated the differentiation between viable (non-stained) and non-viable (blue-stained) cells, thereby quantifying cell death through the uptake of trypan blue dye.

### 4.10. Chromatin Condensation Assay

Apoptosis in Ca9-22 cells was assessed using Hoechst 33342 staining, which highlights nuclear condensation and fragmentation indicative of apoptotic cells [78,97]. Cells were seeded at a density of 10,000 per well in 96-well plates and allowed to adhere for 16 h before being treated with the extract for 24 h. In post-treatment, the cells were rinsed with PBS and stained with Hoechst dye at a concentration of 1 μg/mL in darkness for 10 min. Fluorescence images were captured using the ImageXpress Pico Automated Cell Imaging System (Molecular Devices, San Jose, CA, USA) with DAPI filter cubes. Image acquisition and analysis were performed using CellReporterXpress Version 2 software.

### 4.11. Clonogenic Formation Assay

The proliferation inhibition of Ca9-22 cells was evaluated using a clonogenic formation assay [98]. Cells were seeded at a density of 1000 per well in 6-well plates and allowed to adhere overnight. Afterward, cells were treated with the extract for 5 to 7 days. Cells were then rinsed with PBS, fixed with methanol, and stained with 0.5% crystal violet for 20 min. The number of resultant colonies was counted under a light microscope to assess the degree of proliferation inhibition.

### 4.12. Wound-Healing Assay

The inhibition of Ca9-22 cell migration was assessed using a wound healing assay, a well-established technique to measure collective cell migration on a two-dimensional surface [99]. Initially, cells were grown in serum-reduced medium for six hours to minimize proliferation. A cell-free gap was then introduced into a confluent cell monolayer using a sterile pipette tip. After generating the wound, the cells were rinsed and incubated in serum-reduced medium. The extract was applied, and the cells were observed for 24 h to evaluate their capacity to migrate and close the gap.

### 4.13. Binding Analysis Using AutoDock Vina

Molecular docking interactions between the compounds and the target proteins were assessed using AutoDock Vina [99,100,101,102]. Atomic coordinates for tyrosinase (PDB ID 2Y9X) [55] and *K. pneumoniae* SSB (PDB ID 7F2N) [57] were utilized for the docking simulations. Preparation steps included assigning charges and measuring the active site volumes via AutoDockTools. The two-dimensional structures of the compounds were obtained from PubChem and converted to .sdf format. Both the ligands and protein targets were then prepared in PDBQT format for docking. Virtual screening was performed using the PyRx Virtual Screening Tool, and the docking results were visualized and analyzed with PyMOL software version 2.2.0.

### 4.14. Electrophoretic Mobility Shift Analysis (EMSA)

To analyze the binding of *K. pneumoniae* SSB, a biotinylated deoxythymidine (dT) homopolymer, dT35, was used as a probe. Recombinant *K. pneumoniae* SSB was purified following established protocols [58]. The labeled ssDNA (30 fmol/μL) was incubated with various concentrations of purified *K. pneumoniae* SSB (ranging from 0 to 5000 nM). The electrophoretic mobility shift assay (EMSA) was performed using the LightShift Chemiluminescent EMSA Kit. In this process, *K. pneumoniae* SSB and the DNA were incubated together for 60 min at 37 °C in a reaction buffer containing 40 mM Tris–HCl (pH 7.5) and 50 mM NaCl. After incubation, a loading dye was added, and the samples were loaded onto an 8% native polyacrylamide gel and electrophoresed at 100 V for 1 h in TBE buffer. The resulting protein–DNA complexes were then transferred to a positively charged nylon membrane. The membrane was exposed to UV light at 312 nm for cross-linking. After 10 min of UV exposure, the cross-linked DNA was detected using a streptavidin–horseradish peroxidase conjugate and a chemiluminescent substrate (Pierce Biotechnology, Waltham, MA, USA). The half-maximal protein concentration ([Protein]_50_) necessary to bind 50% of the DNA was calculated from these data.

### 4.15. Inhibition of SSB from K. pneumoniae

*K. pneumoniae* SSB (310 nM) was used in combination with a biotinylated deoxythymidine homopolymer dT35 and the extract at varying concentrations (ranging from 0 to 3000 μg/mL). Following EMSA, the data were used to construct a titration curve. From this, the half-maximal inhibitory concentration (IC_50_) of the extract, which indicates the concentration needed to inhibit 50% of the *K. pneumoniae* SSB activity, was calculated from the curve.

### 4.16. Agar-Well Diffusion Assay

The antibacterial efficacy of the extract was evaluated using the agar-well diffusion method [45]. Pathogens were standardized to a 0.1 McFarland density and spread onto Petri dishes containing 60 mL of Mueller–Hinton agar. The extract, dissolved in 30% DMSO, was applied at a concentration of 10 mg per plate. After incubation at 37 °C for 12 h, the diameter of the inhibition zones (mm) was measured, with larger zones indicating greater antibacterial effectiveness. Ampicillin and 30% DMSO were used as positive and negative controls, respectively. The results presented are the mean standard deviation from a minimum of three separate trials.

### 4.17. Statistical Analysis

Experiments were conducted in triplicate, with results presented as mean ± standard deviation (SD). Statistical significance was determined using GraphPad Prism5 (GraphPad Software Inc., San Diego, CA, USA) with one-way ANOVA employed to assess differences between means.

## 5. Conclusions

This study systematically evaluated the pharmacological properties of acetone-extracted rind from *P. edulis* var. Tainung No. 1, demonstrating its significant therapeutic potential across various applications. The extract exhibited high TPC and TFC, alongside notable antibacterial and anti-SSB activities. It also effectively inhibited key enzymes involved in skin aging, particularly tyrosinase, which plays a pivotal role in melanin production and skin pigmentation disorders. The GC–MS analysis identified several bioactive constituents such as stigmast-5-en-3-ol, vitamin E, palmitic acid, stigmasterol, linoleic acid, campesterol, and octadecanoic acid. Molecular docking studies further supported these compounds as potential dual inhibitors of tyrosinase and SSB, suggesting their roles in both anti-skin aging and antimicrobial activities. The cytotoxic assays revealed that the rind extract suppressed survival, migration, and proliferation of Ca9-22 oral carcinoma cells and induced apoptosis, indicating its potential in cancer therapy, especially against oral carcinoma. Collectively, these findings highlight the substantial medicinal value of *P. edulis* var. Tainung No. 1 rind, typically discarded as agricultural waste, endorsing its utilization as a valuable source of therapeutic agents and promoting sustainable use of agricultural by-products. Future studies should focus on isolating and characterizing specific bioactive compounds and assessing their synergistic effects to advance the development of novel therapeutic agents for clinical applications.

## Figures and Tables

**Table 1:** Anti-skin aging potential of *P. edulis* (Tainung No. 1) rind extracts.

	Inhibition%		
Rind Extract	Tyrosinase	Elastase	Hyaluronidase
Methanol	32.7 ± 2.2	0.0 ± 0.0	15.5 ± 0.4
Ethanol	21.4 ± 1.6	0.0 ± 0.0	11.3 ± 0.3
Acetone	54.5 ± 2.4	0.0 ± 0.0	18.4 ± 0.7
10% DMSO	0.0 ± 0.0	0.0 ± 0.0	0.0 ± 0.0

These experiments utilized the following positive controls: epigallocatechin gallate for anti-elastase activity, which showed 48% inhibition at a concentration of 5 μg/mL; kojic acid for anti-tyrosinase activity, demonstrating 69% inhibition at a concentration of 10 μg/mL; and myricetin for hyaluronidase activity, achieving 49% inhibition at a concentration of 10 μg/mL. Moreover, 10% DMSO served as the negative control.

**Table 2:** Inhibition zone of *P. edulis* (Tainung No. 1) extracts.

	Water	Methanol	Ethanol	Acetone	30% DMSO
*Escherichia coli*	9 ± 2	15 ± 1	13 ± 1	17 ± 2	0 ± 0
*Staphylococcus aureus*	5 ± 1	11 ± 1	9 ± 1	14 ± 2	0 ± 0
*Pseudomonas aeruginosa*	8 ± 1	13 ± 1	11 ± 1	15 ± 1	0 ± 0

Antibacterial efficacy was measured by the diameter of the inhibition zone (mm). Moreover, 30% DMSO served as the negative control. Due to the high amount of extract required for the antibacterial assays, 30% DMSO was necessary to ensure complete dissolution of the extract.

**Table 3:** TPC of *P. edulis* (Tainung No. 1) rind extracts.

	Water	Methanol	Ethanol	Acetone
Rind extract	3.7 ± 0.3	3.8 ± 0.2	3.8 ± 0.2	4.1 ± 0.4

TPC was quantified using the modified Folin–Ciocalteu method. The absorbance of the developed blue color was measured at 750 nm using a UV/VIS spectrophotometer. Results were compared with standard curves of gallic acid (GAE) and expressed as mg gallic acid equivalent per gram of dry weight (mg GAE/g).

**Table 4:** TFC of *P. edulis* (Tainung No. 1) rind extracts.

	Water	Methanol	Ethanol	Acetone
Rind extract	3.7 ± 0.2	4.1 ± 0.3	3.9 ± 0.2	5.4 ± 0.4

TFC was quantified using the aluminum chloride colorimetric method. Absorbance for both extracts and standard solutions was measured at 510 nm. Results were expressed as milligrams of rutin equivalent (RUE) per gram of dry weight (mg RUE/g).

**Table 5:** Binding affinity.

PubChem CID	Compound	Affinity (kcal/mol) to Tyrosinase	Affinity (kcal/mol) to SSB
22012	Stigmast-5-en-3-ol	−7.6	−6.8
14985	Vitamin E	−7.1	−6.1
985	Palmitic acid	−5.0	−5.1
5280794	Stigmasterol	−7.8	−6.8
5280450	Linoleic acid	−5.9	−4.2
173183	Campesterol	−7.6	−6.7
5281	Octadecanoic acid	−5.0	−4.7

**Table 6:** Molecular docking analysis of tyrosinase.

	Interaction	Residue (Distance, Å)
Stigmasterol	Hydrogen bond	Glu322 (4.0)
	Hydrophobic	His85 (3.8), Val248 (3.5), Phe264 (3.6), Phe264 (3.6), Val283 (3.5), Ala286 (3.9)
Campesterol	Hydrogen bond	Ala323 (3.2)
	Hydrophobic	His85 (3.4), Val248 (3.6), His263 (3.9), Phe264 (3.7), Val283 (3.4)
Stigmast-5-en-3-ol	Hydrophobic	His85 (3.5), Asn260 (3.8), His263 (3.8), Phe264 (3.9), Val283 (3.4), Glu322 (3.4), Glu322 (3.8)
Vitamin E	Hydrophobic	Val248 (3.7), Asn260 (4.0), Phe264 (4.0), Phe264 (3.1), Val283 (3.3), Val283 (3.5), Val283 (3.7), Glu322 (4.0)

**Table 7:** Inhibition ability of *P. edulis* rind extracts.

Rind Extract	IC_50_ Value (μg/mL)
Water	>3000
Methanol	958.6 ± 86.0
Ethanol	1261.5 ± 106.8
Acetone	313.2 ± 21.0

IC_50_ values for SSB were determined through the titration curves. The errors are the standard deviation determined at three measurements.

**Table 8:** Molecular docking analysis of SSB.

	Interaction	Residue (Distance, Å)
Stigmasterol	Hydrogen bond	Gln17 * (3.9)
	Hydrophobic	Asn32 * (3.7), Thr34 * (3.7), Arg57 * (3.8), Trp89 * (3.6, 3.7)
Campesterol	Hydrogen bond	Tyr98 * (3.6)
	Hydrophobic	Asn32 (4.0), Trp89 (3.3, 3.6), Lys97 (3.8), Thr99 (3.8)
Stigmast-5-en-3-ol	Hydrogen bond	Thr86 * (3.1)
	Hydrophobic	Thr34 (3.7), Trp55 (3.6, 3.6, 3.7, 3.8, 3.9)
Vitamin E	Hydrophobic	Trp55 (3.4), Arg57 (3.7), Trp89 (3.5), Lys97 (3.6), Tyr98 * (3.6, 3.6, 3.7, 3.9)
	π-Stacking	Tyr98 * (5.0)

* denotes a residue originating from another subunit within the dimer.

## Data Availability

The data are contained within the article.

## Supplementary Materials

The following supporting information can be downloaded at: https://www.mdpi.com/article/10.3390/plants13162194/s1. Figure S1: GC chromatogram. Figure S2: Cytotoxic effect on HEK293T cells.

## References

[1] Atanasov A.G., Zotchev S.B., Dirsch V.M., Supuran C.T. (2021). Natural products in drug discovery: Advances and opportunities. Nat. Rev. Drug Discov..

[2] Jakobušić Brala C., Karković Marković A., Kugić A., Torić J., Barbarić M. (2023). Combination Chemotherapy with Selected Polyphenols in Preclinical and Clinical Studies-An Update Overview. Molecules.

[3] Toma L., Deleanu M., Sanda G.M., Barbălată T., Niculescu L., Sima A.V., Stancu C.S. (2024). Bioactive Compounds Formulated in Phytosomes Administered as Complementary Therapy for Metabolic Disorders. Int. J. Mol. Sci..

[4] Konstantinou E.K., Gioxari A., Dimitriou M., Panoutsopoulos G.I., Panagiotopoulos A.A. (2024). Molecular Pathways of Genistein Activity in Breast Cancer Cells. Int. J. Mol. Sci..

[5] Joma N., Bielawski P.B., Saini A., Kakkar A., Maysinger D. (2024). Nanocarriers for natural polyphenol senotherapeutics. Aging Cell.

[6] Dos Santos F.K.F., Júnior A., Filho A.L.N., Fonseca C.J.N., Isidorio D.K.M., Araújo F.A., Oliveira P.H.A., Veiga Júnior V.F.D. (2024). Graphene and Natural Products: A Review of Antioxidant Properties in Graphene Oxide Reduction. Int. J. Mol. Sci..

[7] Cecerska-Heryć E., Wiśniewska Z., Serwin N., Polikowska A., Goszka M., Engwert W., Michałów J., Pękała M., Budkowska M., Michalczyk A. (2024). Can Compounds of Natural Origin Be Important in Chemoprevention? Anticancer Properties of Quercetin, Resveratrol, and Curcumin-A Comprehensive Review. Int. J. Mol. Sci..

[8] Nikolova K., Velikova M., Gentscheva G., Gerasimova A., Slavov P., Harbaliev N., Makedonski L., Buhalova D., Petkova N., Gavrilova A. (2024). Chemical Compositions, Pharmacological Properties and Medicinal Effects of Genus *Passiflora*, L.: A Review. Plants.

[9] Zhang J., Tao S., Hou G., Zhao F., Meng Q., Tan S. (2023). Phytochemistry, nutritional composition, health benefits and future prospects of Passiflora: A review. Food Chem..

[10] Jenkins G. (2002). Molecular mechanisms of skin ageing. Mech. Ageing Dev..

[11] Dańczak-Pazdrowska A., Gornowicz-Porowska J., Polańska A., Krajka-Kuźniak V., Stawny M., Gostyńska A., Rubiś B., Nourredine S., Ashiqueali S., Schneider A. (2023). Cellular senescence in skin-related research: Targeted signaling pathways and naturally occurring therapeutic agents. Aging Cell.

[12] Rathore G., Das K., Landau M., Verner I., Kassir M., Galadari H.I., Gold M.H., Babaei M., Goldust M. (2024). Clinical Assessment, Diagnosis, and Management of Infraorbital Wrinkles and Pigmentation. Dermatol. Clin..

[13] Goh C.F. (2024). Diversity of Asian skin: A review on skin biophysical properties. Exp. Dermatol..

[14] Michalak M. (2023). Plant Extracts as Skin Care and Therapeutic Agents. Int. J. Mol. Sci..

[15] Zhang S., Duan E. (2018). Fighting against Skin Aging: The Way from Bench to Bedside. Cell Transplant..

[16] Orqueda M.E., Zampini I.C., Bravo K., Osorio E., Isla M.I. (2022). Potential use of native fruits waste from Argentina as nonconventional sources of cosmetic ingredients. J. Cosmet. Dermatol..

[17] Srikanya S., Shiesh C.C., Lin H.L., Julius I.P. (2014). Growth and Development of ‘Tainung NO.1’ Passion Fruit (*Passiflora edulis* Sims) in Taiwan. Horticulture NCHU.

[18] Darby E.M., Trampari E., Siasat P., Gaya M.S., Alav I., Webber M.A., Blair J.M.A. (2023). Molecular mechanisms of antibiotic resistance revisited. Nat. Rev. Microbiol..

[19] Du Toit A. (2022). The cost of resistance. Nat. Rev. Microbiol..

[20] Tommasi R., Brown D.G., Walkup G.K., Manchester J.I., Miller A.A. (2015). ESKAPEing the labyrinth of antibacterial discovery. Nat. Rev. Drug Discov..

[21] Klevens R.M., Morrison M.A., Nadle J., Petit S., Gershman K., Ray S., Harrison L.H., Lynfield R., Dumyati G., Townes J.M. (2007). Invasive methicillin-resistant Staphylococcus aureus infections in the United States. JAMA.

[22] Kokoska L., Kloucek P., Leuner O., Novy P. (2019). Plant-Derived Products as Antibacterial and Antifungal Agents in Human Health Care. Curr. Med. Chem..

[23] Bush K. (2010). Alarming beta-lactamase-mediated resistance in multidrug-resistant Enterobacteriaceae. Curr. Opin. Microbiol..

[24] Zhao W.H., Hu Z.Q. (2010). Beta-lactamases identified in clinical isolates of Pseudomonas aeruginosa. Crit. Rev. Microbiol..

[25] Bonde N.J., Kozlov A.G., Cox M.M., Lohman T.M., Keck J.L. (2024). Molecular insights into the prototypical single-stranded DNA-binding protein from *E. coli*. Crit. Rev. Biochem. Mol. Biol..

[26] Antony E., Lohman T.M. (2019). Dynamics of *E. coli* single stranded DNA binding (SSB) protein-DNA complexes. Semin. Cell Dev. Biol..

[27] Shereda R.D., Kozlov A.G., Lohman T.M., Cox M.M., Keck J.L. (2008). SSB as an organizer/mobilizer of genome maintenance complexes. Crit. Rev. Biochem. Mol. Biol..

[28] Lohman T.M., Ferrari M.E. (1994). Escherichia coli single-stranded DNA-binding protein: Multiple DNA-binding modes and cooperativities. Annu. Rev. Biochem..

[29] Bianco P.R. (2021). The mechanism of action of the SSB interactome reveals it is the first OB-fold family of genome guardians in prokaryotes. Protein Sci..

[30] Tan H.Y., Wilczek L.A., Pottinger S., Manosas M., Yu C., Nguyenduc T., Bianco P.R. (2017). The intrinsically disordered linker of E. coli SSB is critical for the release from single-stranded DNA. Protein Sci..

[31] Bianco P.R., Pottinger S., Tan H.Y., Nguyenduc T., Rex K., Varshney U. (2017). The IDL of *E. coli* SSB links ssDNA and protein binding by mediating protein-protein interactions. Protein Sci..

[32] Bianco P.R. (2017). The tale of SSB. Prog. Biophys. Mol. Biol..

[33] Yu C., Tan H.Y., Choi M., Stanenas A.J., Byrd A.K., Raney K.D., Cohan C.S., Bianco P.R. (2016). SSB binds to the RecG and PriA helicases in vivo in the absence of DNA. Genes. Cells.

[34] Huang C.Y. (2018). Crystal structure of SSB complexed with inhibitor myricetin. Biochem. Biophys. Res. Commun..

[35] Voter A.F., Killoran M.P., Ananiev G.E., Wildman S.A., Hoffmann F.M., Keck J.L. (2017). A High-Throughput Screening Strategy to Identify Inhibitors of SSB Protein-Protein Interactions in an Academic Screening Facility. SLAS Discov..

[36] Glanzer J.G., Endres J.L., Byrne B.M., Liu S., Bayles K.W., Oakley G.G. (2016). Identification of inhibitors for single-stranded DNA-binding proteins in eubacteria. J. Antimicrob. Chemother..

[37] Boucher H.W., Talbot G.H., Bradley J.S., Edwards J.E., Gilbert D., Rice L.B., Scheld M., Spellberg B., Bartlett J. (2009). Bad bugs, no drugs: No ESKAPE! An update from the Infectious Diseases Society of America. Clin. Infect. Dis..

[38] Dong N., Yang X., Chan E.W., Zhang R., Chen S. (2022). Klebsiella species: Taxonomy, hypervirulence and multidrug resistance. EBioMedicine.

[39] Gil G.F., Anderson J.A., Aravkin A., Bhangdia K., Carr S., Dai X., Flor L.S., Hay S.I., Malloy M.J., McLaughlin S.A. (2024). Health effects associated with chewing tobacco: A Burden of Proof study. Nat. Commun..

[40] Johnson D.E., Burtness B., Leemans C.R., Lui V.W.Y., Bauman J.E., Grandis J.R. (2020). Head and neck squamous cell carcinoma. Nat. Rev. Dis. Prim..

[41] Cramer J.D., Burtness B., Le Q.T., Ferris R.L. (2019). The changing therapeutic landscape of head and neck cancer. Nat. Rev. Clin. Oncol..

[42] Panarese I., Aquino G., Ronchi A., Longo F., Montella M., Cozzolino I., Roccuzzo G., Colella G., Caraglia M., Franco R. (2019). Oral and Oropharyngeal squamous cell carcinoma: Prognostic and predictive parameters in the etiopathogenetic route. Expert Rev. Anticancer Ther..

[43] Contrera K.J., Zafereo M.E., Yaniv D., Roberts D.B., Gillenwater A.M., Hanna E.Y., Weber R.S., Myers J.N., Chang E.I., Garvey P.B. (2022). Outcomes for recurrent oral cavity squamous cell carcinoma. Oral Oncol..

[44] Nandini D.B., Rao R.S., Hosmani J., Khan S., Patil S., Awan K.H. (2020). Novel therapies in the management of oral cancer: An update. Dis. Mon..

[45] Balouiri M., Sadiki M., Ibnsouda S.K. (2016). Methods for in vitro evaluating antimicrobial activity: A review. J. Pharm. Anal..

[46] Fraga C.G., Croft K.D., Kennedy D.O., Tomas-Barberan F.A. (2019). The effects of polyphenols and other bioactives on human health. Food Funct..

[47] Flieger J., Raszewska-Famielec M., Radzikowska-Büchner E., Flieger W. (2024). Skin Protection by Carotenoid Pigments. Int. J. Mol. Sci..

[48] Đurović S., Kojić I., Radić D., Smyatskaya Y.A., Bazarnova J.G., Filip S., Tosti T. (2024). Chemical Constituents of Stinging Nettle (*Urtica dioica* L.): A Comprehensive Review on Phenolic and Polyphenolic Compounds and Their Bioactivity. Int. J. Mol. Sci..

[49] Li J.W., Vederas J.C. (2009). Drug discovery and natural products: End of an era or an endless frontier?. Science.

[50] Baur J.A., Sinclair D.A. (2006). Therapeutic potential of resveratrol: The in vivo evidence. Nat. Rev. Drug Discov..

[51] Ross J.A., Kasum C.M. (2002). Dietary flavonoids: Bioavailability, metabolic effects, and safety. Annu. Rev. Nutr..

[52] Naranjo-Durán A.M., Quintero-Quiroz J., Ciro-Gómez G.L., Barona-Acevedo M.J., Contreras-Calderón J.C. (2023). Characterization of the antioxidant activity, carotenoid profile by HPLC-MS of exotic colombian fruits (goldenberry and purple passion fruit) and optimization of antioxidant activity of this fruit blend. Heliyon.

[53] Logesh R., Prasad S.R., Chipurupalli S., Robinson N., Mohankumar S.K. (2023). Natural tyrosinase enzyme inhibitors: A path from melanin to melanoma and its reported pharmacological activities. Biochim. Biophys. Acta Rev. Cancer.

[54] Baber M.A., Crist C.M., Devolve N.L., Patrone J.D. (2023). Tyrosinase Inhibitors: A Perspective. Molecules.

[55] Ismaya W.T., Rozeboom H.J., Weijn A., Mes J.J., Fusetti F., Wichers H.J., Dijkstra B.W. (2011). Crystal structure of Agaricus bisporus mushroom tyrosinase: Identity of the tetramer subunits and interaction with tropolone. Biochemistry.

[56] Huang Y.H., Guan H.H., Chen C.J., Huang C.Y. (2017). Staphylococcus aureus single-stranded DNA-binding protein SsbA can bind but cannot stimulate PriA helicase. PLoS ONE.

[57] Lin E.S., Huang Y.H., Huang C.Y. (2021). Characterization of the Chimeric PriB-SSBc Protein. Int. J. Mol. Sci..

[58] Huang Y.H., Huang C.Y. (2012). Characterization of a single-stranded DNA-binding protein from *Klebsiella pneumoniae*: Mutation at either Arg73 or Ser76 causes a less cooperative complex on DNA. Genes Cells.

[59] Jan H.C., Lee Y.L., Huang C.Y. (2011). Characterization of a single-stranded DNA-binding protein from *Pseudomonas aeruginosa* PAO1. Protein J..

[60] Huang Y.H., Lee Y.L., Huang C.Y. (2011). Characterization of a single-stranded DNA binding protein from *Salmonella enterica* serovar Typhimurium LT2. Protein J..

[61] Huang Y.H., Lin E.S., Huang C.Y. (2019). Complexed crystal structure of SSB reveals a novel single-stranded DNA binding mode (SSB)3:1: Phe60 is not crucial for defining binding paths. Biochem. Biophys. Res. Commun..

[62] Huang Y.H., Chen I.C., Huang C.Y. (2019). Characterization of an SSB-dT25 complex: Structural insights into the S-shaped ssDNA binding conformation. RSC Adv..

[63] Chang S.K., Alasalvar C., Shahidi F. (2019). Superfruits: Phytochemicals, antioxidant efficacies, and health effects—A comprehensive review. Crit. Rev. Food Sci. Nutr..

[64] Lee K.W., Bode A.M., Dong Z. (2011). Molecular targets of phytochemicals for cancer prevention. Nat. Rev. Cancer.

[65] Papaemmanouil C.D., Peña-García J., Banegas-Luna A.J., Kostagianni A.D., Gerothanassis I.P., Pérez-Sánchez H., Tzakos A.G. (2022). ANTIAGE-DB: A Database and Server for the Prediction of Anti-Aging Compounds Targeting Elastase, Hyaluronidase, and Tyrosinase. Antioxidants.

[66] Dzialo M., Mierziak J., Korzun U., Preisner M., Szopa J., Kulma A. (2016). The Potential of Plant Phenolics in Prevention and Therapy of Skin Disorders. Int. J. Mol. Sci..

[67] Chen Q.X., Kubo I. (2002). Kinetics of mushroom tyrosinase inhibition by quercetin. J. Agric. Food Chem..

[68] Zolghadri S., Beygi M., Mohammad T.F., Alijanianzadeh M., Pillaiyar T., Garcia-Molina P., Garcia-Canovas F., Munoz-Munoz J., Saboury A.A. (2023). Targeting tyrosinase in hyperpigmentation: Current status, limitations and future promises. Biochem. Pharmacol..

[69] Nagatsu T., Nakashima A., Watanabe H., Ito S., Wakamatsu K. (2022). Neuromelanin in Parkinson’s Disease: Tyrosine Hydroxylase and Tyrosinase. Int. J. Mol. Sci..

[70] Bose A., Petsko G.A., Eliezer D. (2018). Parkinson’s Disease and Melanoma: Co-Occurrence and Mechanisms. J. Parkinsons Dis..

[71] Lleó A., Greenberg S.M., Growdon J.H. (2006). Current pharmacotherapy for Alzheimer’s disease. Annu. Rev. Med..

[72] Bottiglieri T., Arning E., Wasek B., Nunbhakdi-Craig V., Sontag J.M., Sontag E. (2012). Acute administration of L-DOPA induces changes in methylation metabolites, reduced protein phosphatase 2A methylation, and hyperphosphorylation of Tau protein in mouse brain. J. Neurosci..

[73] Iannitelli A.F., Weinshenker D. (2023). Riddles in the dark: Decoding the relationship between neuromelanin and neurodegeneration in locus coeruleus neurons. Neurosci. Biobehav. Rev..

[74] Lin E.S., Luo R.H., Huang C.Y. (2022). A Complexed Crystal Structure of a Single-Stranded DNA-Binding Protein with Quercetin and the Structural Basis of Flavonol Inhibition Specificity. Int. J. Mol. Sci..

[75] Lin E.S., Huang Y.H., Luo R.H., Basharat Z., Huang C.Y. (2022). Crystal Structure of an SSB Protein from Salmonella enterica and Its Inhibition by Flavanonol Taxifolin. Int. J. Mol. Sci..

[76] Maura D., Ballok A.E., Rahme L.G. (2016). Considerations and caveats in anti-virulence drug development. Curr. Opin. Microbiol..

[77] Lin E.S., Huang Y.H., Chung J.C., Su H.H., Huang C.Y. (2023). The Inhibitory Effects and Cytotoxic Activities of the Stem Extract of Nepenthes miranda against Single-Stranded DNA-Binding Protein and Oral Carcinoma Cells. Plants.

[78] Liu H.W., Chiang W.Y., Huang Y.H., Huang C.Y. (2022). The Inhibitory Effects and Cytotoxic Activities of the Stem Extract of *Sarracenia purpurea* against Melanoma Cells and the SsbA Protein. Plants.

[79] Sugino A., Peebles C.L., Kreuzer K.N., Cozzarelli N.R. (1977). Mechanism of action of nalidixic acid: Purification of *Escherichia coli* nalA gene product and its relationship to DNA gyrase and a novel nicking-closing enzyme. Proc. Natl. Acad. Sci. USA.

[80] Gellert M., O’Dea M.H., Itoh T., Tomizawa J. (1976). Novobiocin and coumermycin inhibit DNA supercoiling catalyzed by DNA gyrase. Proc. Natl. Acad. Sci. USA.

[81] Fernando I.P.S., Sanjeewa K.K.A., Ann Y.S., Ko C.I., Lee S.H., Lee W.W., Jeon Y.J. (2018). Apoptotic and antiproliferative effects of Stigmast-5-en-3-ol from *Dendronephthya gigantea* on human leukemia HL-60 and human breast cancer MCF-7 cells. Toxicol. In Vitro.

[82] Pang K.L., Mai C.W., Chin K.Y. (2023). Molecular Mechanism of Tocotrienol-Mediated Anticancer Properties: A Systematic Review of the Involvement of Endoplasmic Reticulum Stress and Unfolded Protein Response. Nutrients.

[83] Zhu S., Jiao W., Xu Y., Hou L., Li H., Shao J., Zhang X., Wang R., Kong D. (2021). Palmitic acid inhibits prostate cancer cell proliferation and metastasis by suppressing the PI3K/Akt pathway. Life Sci..

[84] Miszczuk E., Bajguz A., Kiraga Ł., Crowley K., Chłopecka M. (2024). Phytosterols and the Digestive System: A Review Study from Insights into Their Potential Health Benefits and Safety. Pharmaceuticals.

[85] Khan A.U., Khan A., Shal B., Khan S., Khan M., Ahmad R., Riaz M. (2023). The critical role of the phytosterols in modulating tumor microenvironment via multiple signaling: A comprehensive molecular approach. Phytother. Res..

[86] Mishra V., Tomar S., Yadav P., Singh M.P. (2021). Promising anticancer activity of polysaccharides and other macromolecules derived from oyster mushroom (*Pleurotus* sp.): An updated review. Int. J. Biol. Macromol..

[87] Mustafa A.M., Abouelenein D., Acquaticci L., Alessandroni L., Angeloni S., Borsetta G., Caprioli G., Nzekoue F.K., Sagratini G., Vittori S. (2022). Polyphenols, Saponins and Phytosterols in Lentils and Their Health Benefits: An Overview. Pharmaceuticals.

[88] Singleton V.L., Orthofer R., Lamuela-Raventos R.M. (1999). Analysis of total phenols and other oxidation substrates and antioxidants by means of folin-ciocalteu reagent. Methods Enzymol..

[89] Huang C.Y. (2015). Inhibition of a putative dihydropyrimidinase from *Pseudomonas aeruginosa* PAO1 by flavonoids and substrates of cyclic amidohydrolases. PLoS ONE.

[90] Chang C.C., Yang M.H., Wen H.M., Chern J.C. (2002). Estimation of total flavonoid content in propolis by two complementary colorimetric methods. J. Food Drug Anal..

[91] Lee C.Y., Chen Y.C., Huang Y.H., Lien Y., Huang C.Y. (2024). Cytotoxicity and Multi-Enzyme Inhibition of Nepenthes miranda Stem Extract on H838 Human Non-Small Cell Lung Cancer Cells and RPA32, Elastase, Tyrosinase, and Hyaluronidase Proteins. Plants.

[92] Shimizu K., Kondo R., Sakai K., Lee S.H., Sato H. (1998). The inhibitory components from Artocarpus incisus on melanin biosynthesis. Planta Med..

[93] Tu P.T., Tawata S. (2015). Anti-Oxidant, Anti-Aging, and Anti-Melanogenic Properties of the Essential Oils from Two Varieties of *Alpinia zerumbet*. Molecules.

[94] Kim Y.J., Uyama H., Kobayashi S. (2004). Inhibition effects of (+)-catechin-aldehyde polycondensates on proteinases causing proteolytic degradation of extracellular matrix. Biochem. Biophys. Res. Commun..

[95] Guan H.H., Huang Y.H., Lin E.S., Chen C.J., Huang C.Y. (2021). Plumbagin, a Natural Product with Potent Anticancer Activities, Binds to and Inhibits Dihydroorotase, a Key Enzyme in Pyrimidine Biosynthesis. Int. J. Mol. Sci..

[96] Strober W. (2015). Trypan blue exclusion test of cell viability. Curr. Protoc. Immunol..

[97] Larsson R., Nygren P. (1989). A rapid fluorometric method for semiautomated determination of cytotoxicity and cellular proliferation of human tumor cell lines in microculture. Anticancer Res..

[98] Chen M.H., Yang W.L., Lin K.T., Liu C.H., Liu Y.W., Huang K.W., Chang P.M., Lai J.M., Hsu C.N., Chao K.M. (2011). Gene expression-based chemical genomics identifies potential therapeutic drugs in hepatocellular carcinoma. PLoS ONE.

[99] Liang C.C., Park A.Y., Guan J.L. (2007). In vitro scratch assay: A convenient and inexpensive method for analysis of cell migration in vitro. Nat. Protoc..

[100] Dallakyan S., Olson A.J. (2015). Small-molecule library screening by docking with PyRx. Methods Mol. Biol..

[101] Trott O., Olson A.J. (2010). AutoDock Vina: Improving the speed and accuracy of docking with a new scoring function, efficient optimization, and multithreading. J. Comput. Chem..

[102] Morris G.M., Huey R., Lindstrom W., Sanner M.F., Belew R.K., Goodsell D.S., Olson A.J. (2009). AutoDock4 and AutoDockTools4: Automated docking with selective receptor flexibility. J. Comput. Chem..

