# Supplementary material for: Anti-Skin Aging Potential, Antibacterial Activity, Inhibition of Single-Stranded DNA-Binding Protein, and Cytotoxic Effects of Acetone-Extracted Passiflora edulis (Tainung No. 1) Rind Extract on Oral Carcinoma Cells
Source: Plants (Basel). 2024 Aug 8;13(16):2194. doi: 10.3390/plants13162194 (PMC11359509; doi:10.3390/plants13162194)
Supplement: Supplementary file 1 [file plants-13-02194-s001.zip › plants-3126900-supplementary.pdf]

Supplementary Material: Figure S1.

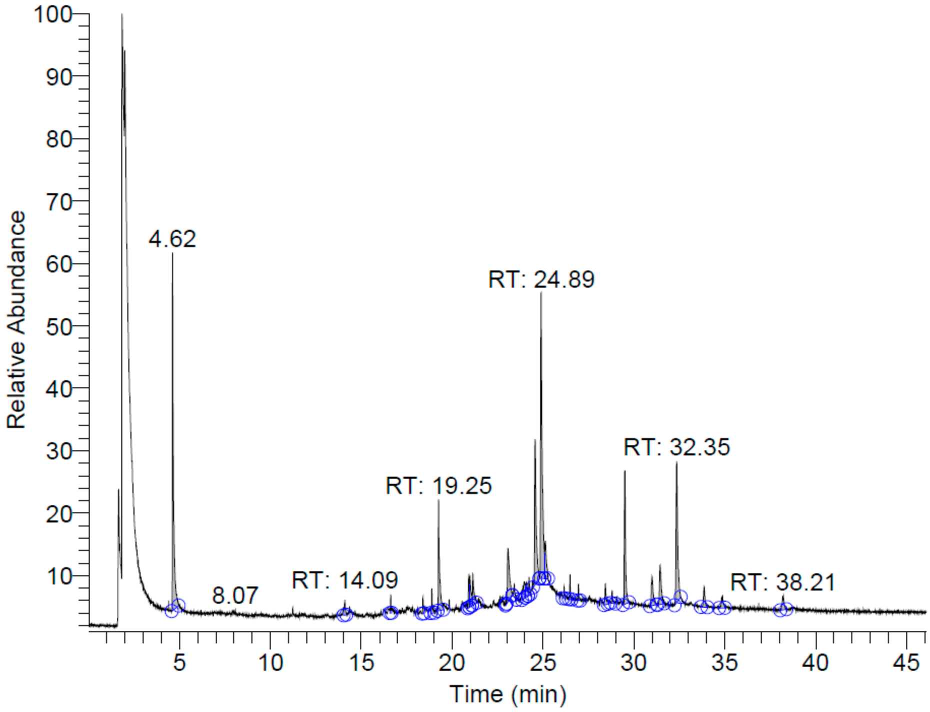

Figure S1. GC chromatogram. The content within the acetone-extracted rind extract was analyzed by GC–MS. By matching the generated spectra with mass spectral libraries, compounds were tentatively identified.

Supplementary Material: Figure S2.

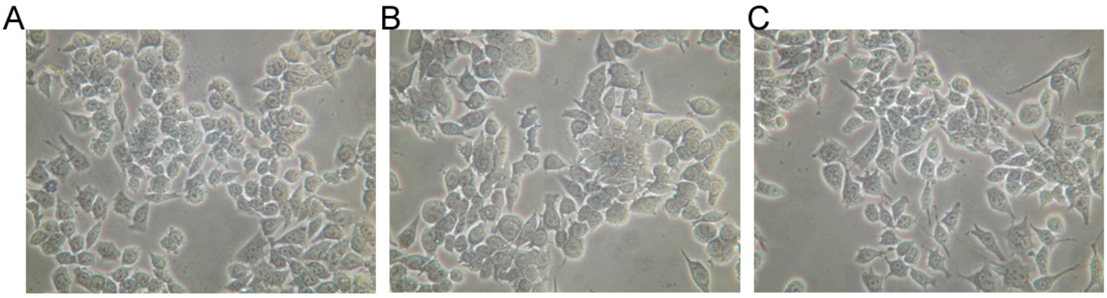

Figure S2. Cytotoxic effect on HEK293T cells. Results from the trypan blue exclusion assay show that treatment with extract concentrations of (A) 0, (B) 1000, and (C) 1250 µg/mL did not induce death in HEK293T cells.
